# On the Influence of Cold Applied to the Scrotum, in Restraining Bleeding from the Nose

**Published:** 1809-10

**Authors:** Robert Kinglake

**Affiliations:** Taunton


### *On the Influence of Cold applied to the Scrotum, in restraining Bleeding from the Nose.* By Dr. Kinglake.

A Case sometime since occurred in this place, of a middle aged man, who suffered under a protracted and unyielding bleeding from the nose. The usual modes of treatment were unsuccessfully instituted, the patient became daily more and more exhausted, until life itself appeared to be eminently endangered. Under these circumstances, it was proposed, that the restrictive influence of suddenly applying cold water to the region of the genitals, more particularly to the scrotum, should be tried. This was done by means of a large folded cloth wetted in that fluid, which induced a sense of strong shudder throughout the frame, and almost immediately restrained the bleeding. Nor was the effect transient; no further effusion of blood occurred.

The salutary influence of cold applied to the genital parts in bleeding at the nose, has not been discovered by recent experience. At so remote a period as in the time of Diemerbroeck, nearly 150 years since, it was successfully employed. Diemerbroeck records the efficacy of the remedy in the following words; speaking of hæmorrhage from the nose, he says, "A gravi casu vena in nasibus disrupta et operta fuit, hincque sanguis affatim et impetuose largo ductu effluxit haud minus quam si mediana brachii incisa fuisset. Oxycratum cervici et collo circumjectum, nasibus quoque et fronte applicatum, atque in nares infusum fuit, ligaturæ extremarum partium etiam injectæ sed omnia hæc nihil profuerunt. Interea æger ab ingenti sanguinis effluentis copia, animo linquebatur. Cum autem reliqua ista nihil juvarent, nec chirurgus qui venam secaret ad manus esset, mantile quadruplicatum aquæ frigidæ immergi jussi ac testibus circumponi quod cùm bis reiteratum esset, præter astantium spinionem hæmorrhagia substitit, et æger ad se rediit.—Observatio xi.—Opera Omnia."

The sudden application of cold to the forehead, neck, and between the shoulders, in cases of obstinate bleeding at the nose, is a familiar and often an efficacious practice; but it has been more rare to prefer the region of the genitals as best suited to give full effect to its beneficial operation. The principle on which it acts, appears to be that of reducing within diminished limits, the dilating influence of vital heat, by which a general contraction is induced throughout the sanguiferous system, and thus any accidental accidental aperture or vessel from which blood might be effusing may be effectually closed. As the contracting effect on this occasion must be proportionate to the intensity of impression made by the application of cold, it is but just to admit, that it must be stronger when brought into contact with the genital parts, than with any other situation, in as far as these parts exceed in degree of temperature and sensibility, what is found to exist in any other portion of the system. It is therefore reasonable to conclude, that both the shock of the impression, and the consequent contractile influence will be proportionately greater, and in the case referred to, will prove more efficacious than if the remedy were to be applied to any other part of the cuticular surface.

It may be justly inferred from fair analogy, that if topical cold on the genitals should be more influential on the action of the sanguiferous system, than when applied to any other part, it would be equally availing in threatening cases of menorrhagia, hæmatemesis, hæmaturia, hæmoptysis, hæmorrhois, &c. The remedy is simple, and of easy application; and if its reputed efficacy should prove to be well founded, it is not unworthy of professional adoption.

I am, &c.

ROBERT KINGLAKE.

*Taunton, September* 14, 1809.

